# Chronic *Toxoplasma gondii* infection and sleep‐wake alterations in mice

**DOI:** 10.1111/cns.13650

**Published:** 2021-06-04

**Authors:** Damien Dupont, Jian‐Sheng Lin, François Peyron, Hideo Akaoka, Martine Wallon

**Affiliations:** ^1^ Institut des Agents Infectieux Parasitologie Mycologie Hôpital de la Croix‐Rousse Hospices Civils de Lyon Lyon France; ^2^ Physiologie intégrée du système d’éveil Faculté de Médecine Centre de Recherche en Neurosciences de Lyon INSERM U1028‐CNRS UMR 5292 Université Claude Bernard Lyon 1 Lyon France

**Keywords:** infection, neuropsychiatric disorders, sleep, *Toxoplasma*, wakefulness

## Abstract

**Aim:**

*Toxoplasma gondii* (*Tg*) is an intracellular parasite infecting more than a third of the human population. Yet, the impact of *Tg* infection on sleep, a highly sensitive index of brain functions, remains unknown. We designed an experimental mouse model of chronic *Tg* infection to assess the effects on sleep‐wake states.

**Methods:**

Mice were infected using cysts of the type II Prugniaud strain. We performed chronic sleep‐wake recordings and monitoring as well as EEG power spectral density analysis in order to assess the quantitative and qualitative changes of sleep‐wake states. Pharmacological approach was combined to evaluate the direct impact of the infection and inflammation caused by *Tg*.

**Results:**

Infected mouse exhibited chronic sleep‐wake alterations over months, characterized by a marked increase (>20%) in time spent awake and in cortical EEG θ power density of all sleep‐wake states. Meanwhile, slow‐wave sleep decreased significantly. These effects were alleviated by an anti‐inflammatory treatment using corticosteroid dexamethasone.

**Conclusion:**

We demonstrated for the first time the direct consequences of *Tg* infection on sleep‐wake states. The persistently increased wakefulness and reduced sleep fit with the parasite's strategy to enhance dissemination through host predation and are of significance in understanding the neurodegenerative and neuropsychiatric disorders reported in infected patients.

## Graphical abstract

When mice are infected chronically by *Toxoplasma gondii*, an intracellular parasite, they exhibit a marked increase in wakefulness and activities, thus promoting encounters with cats, its natural predators. It is likely that by this strategy to modify its hosts’ sleep‐wake and behavioral states, *Toxoplasma gondii* would enhance its own dissemination, thus explaining why it infects a wide range of animals and about one third of the world population.

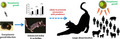

**Abbreviations**
DXM: dexamethasone
EEG: electroencephalogram
EMG: electromyogram
pi: post‐infection
PS: paradoxical sleep
SWS: slow‐wave sleep
SXT: Sulfamethoxazole‐Trimethoprim
*Tg*
: *Toxoplasma gondii*
WK: waking

## 1 INTRODUCTION

*Toxoplasma gondii* (*Tg*) is a remarkably successful obligate intracellular protozoan parasite that infects all warm‐blooded animals and 30% of the world population.^1^ After infection, the parasite transforms into fast replicating tachyzoites that disseminate throughout the host's tissues. Subsequent development of cellular immunity and production of antibodies lead the parasite into entering a chronic phase whereby it remains quiescent for the life of the host as tissue cysts with bradyzoite stages preferentially located in the central nervous system (CNS), eyes, and skeletal muscles.^2^

Accumulated evidence indicates that this persistence of *Tg* may lead to physiological and behavioral consequences in infected rodents, resulting in reduced fear of Felidae their natural predators and even sexual attraction to their urine,^3^, ^4^, ^5^, ^6^, ^7^, ^8^, ^9^ as well as decreased mechanisms of warning and anxiety,^10^ and higher activity level.^11^

*Tg* infection may also contribute to neurological and psychiatric symptoms in humans. Epidemiological studies suggested a possible link between *Tg* infection and schizophrenia^12^, ^13^ and other mood, behavioral, or neurological disorders. Several literature reviews confirmed the heterogeneity of designs and findings and failed to provide clear conclusions. Yet, these human studies have provided interesting clues, contradicting the current belief that chronic postnatal infection is asymptomatic in immune‐competent subjects.

Surprisingly, little attention has been paid to sleep‐wake cycle alteration. Yet, the large prevalence of sleep disorders in the general population closely matches that of toxoplasmosis, both estimated to be around 30%.^1^, ^14^ Moreover, sleep constitutes a highly sensitive index of brain functions, as sleep disorders are often found to be associated with mood, behavioral, and neurological disorders, even at their early stage.^15^, ^16^, ^17^ Interestingly, *Tg* cysts are randomly distributed in all brain areas, but overlapping those that control the sleep‐wake cycle.^18^, ^19^ Of note, neuromodulators known as sleep‐wake regulators, such as melatonin and monoamines including norepinephrine, dopamine, and serotonin, are found to be markedly altered by *Tg* infection, indicating that it can affect vigilance states.^20^, ^21^, ^22^ Finally, our clinical records indicate occurrence of sleep disturbances in a cohort of congenitally and postnatally infected individuals (unpublished data).

We therefore sought to establish a mouse model to unambiguously assess the consequences of chronic *Tg* infection on sleep and wakefulness in laboratory‐controlled conditions, and thus, to highlight the impact of sleep alteration on behavioral, neurological, and neuropsychological functions in infected patients and to provide the required guidance for human studies.

## 2 MATERIAL AND METHODS

### 2.1 Animals

All experiments were conducted according to the European Ethics Community Directive for the use of research animals (2010/63/EC). We followed the 3R principles to minimize the number of animals used, pain, and discomfort. The experimental protocol and procedures were approved by the local ethics committee for animal experimentation of Lyon 1 University (No. APAFIS#22363‐2019100915103240). Both experimental design and conduct of the study were performed strictly according to the ARRIVE guidelines.^23^

Male, inbred CBA/Jrj mice were purchased from Janvier Labs, France with a certification as free of pathogenic viral, bacterial, and parasitic contaminants. Known to be susceptible to chronic infection by Tg, they were housed two per cage in our BSL2 animal facility (Hôpital de la Croix‐Rousse, Lyon, France). Room temperature was maintained at 22°C (+/‐ 2°C) with a 12 h light/dark cycle (light‐on at 7am). Animals were monitored every day and handled regularly for habituation.

### 2.2 Infection with *Tg*

All procedures manipulating infectious materials were performed in our BSL2 animal facility. Outbred female OF1 mice were also purchased from Janvier Labs, France. They were housed four per cage and were only used to multiply and perpetuate the *Tg* strain used for inoculation. We used *Tg* cysts of the canonical type II, avirulent PRU strain, representative of the European epidemiology. Considering its kystogenic profile, it allows to study the chronic phase of toxoplasmosis. We obtained the initial batch (Strain TgH 00001) from the French National Reference Center, as a brain homogenate of female Swiss Webster mice that had been infected intraperitoneally. The homogenate was diluted in sterile saline. OF1 mice were chronically infected by intraperitoneal injection of five cysts per mouse to maintain the *Tg* strain. On the day of inoculation, infected OF1 mice were euthanized under deep anesthesia. Their brains were removed, then homogenized in sterile saline. Using this homogenate, we orally infected 11‐ to 14‐week‐old CBA/Jrj mice by gavage (20 cysts) to mimic the natural route of infection and avoid possible peritoneal inflammation.^24^ Control CBA/Jrj mice were inoculated in the same fashion with a brain homogenate prepared from healthy, non‐infected OF1 mice. In order to study the chronic phase of infection, inoculated and control mice underwent surgery as described below between 18 and 22 weeks of age—at least eight weeks after inoculation—and with a body weight of 25‐35 g. Infection was verified by the presence of blood‐borne anti‐*Tg* antibodies, using an ELISA kit (bioMérieux, France), between 4 and 5 weeks post‐infection (pi).

### 2.3 Surgery

Control and infected CBA/Jrj mice were anesthetized using isoflurane (oxygen flow rate 200 mL/min, 3%‐4% initially, maintenance at 1%). We placed each mouse in a stereotaxic apparatus with a heating pad maintaining the animal rectal temperature at 37 ± 1°C. After shaving, the skin over the skull was rubbed with iodine, sectioned longitudinally, and reclined to expose the skull. Four holes (Ø = 0.5 mm) were drilled in the skull at the following coordinates: frontal (1 mm lateral and anterior to bregma) and parietal (1 mm lateral to the midline at the midpoint between bregma and lambda) cortices of both left and right sides. These procedures preserve the integrity of all brain‐protecting membranes (dura and pia, particularly). Four cortical electrodes (gold‐plated, tinned‐copper wire, Ø = 0.4 mm; Filotex, Draveil, France) were placed into these holes for electroencephalogram **(**EEG). Three muscle electrodes (gold‐plated fluorocarbon‐coated stainless‐steel wire, Ø = 0.03 mm; Cooner Wire, Chatworth, CA) were inserted into the neck for electromyogram (EMG) recordings. EEG was differentially recorded between two electrodes overlying frontal and parietal cortice of one side. These electrodes were priorly soldered to a multichannel electrical connector, and each was separately insulated with heat‐shrinkable polyolefin/polyester tubing. Finally, the electrode assembly was anchored to the skull with Super‐Bond (Sun Medical Co., Shiga, Japan) and embedded with dental cement. The skin was sutured around implant. After subcutaneous injections of Carprofen (Rimadyl, 5 mg/kg) and sterile saline containing 5% glucose, animals were warmed until recovery. We kept each mouse under close observation for another six days: weight, behavior, eating, and drinking activities were monitored. Our implantation procedures allowed stable polygraphic recordings for more than six months.

### 2.4 Polysomnographic data acquisition and analysis

After surgery, each mouse was housed individually in a transparent barrel (Ø 20 cm, height 30 cm) in a ventilated animal cabinet located in a sound‐proof recording room with an ambient temperature of 22 ± 2°C, a 12 h light/dark cycle (light‐on at 7am), food and water *ad libitum*. Mice were habituated to the recording cable connecting implant to a swivel connector for five days before starting polysomnographic (EEG and EMG) recordings.

Fronto‐parietal EEG and EMG signals were amplified and filtered (bandwidth 0.3‐100 Hz for EEG, 10‐100 Hz for EMG). These signals were digitalized with CED 1401 converter (Cambridge Electronic Design, UK) with a sampling rate of 512 Hz using Spike2 software (Cambridge Electronic Design). We used Sleepscore software (ViewPoint, Lyon, France) to determine the vigilance states every 5‐sec epoch: WK, SWS, and PS, according to previously described criteria.^25^, ^26^, ^27^ All sleep‐wake recordings were scored blind. Total durations (±SEM) of each vigilance state were calculated for light (7am to 7 pm), dark (7 pm to 7am), and 24 h periods. For each vigilance state, the total number of episodes and the mean duration (±SEM) of one episode were calculated for each period. All durations were expressed in minutes. In order to evaluate the contrast of sleep‐wake states between light and dark phases and as a criterion of sleep‐wake circadian rhythms, we used the light/dark ratio for sleep stages (dark/light for WK), obtained by 12 hours sleep‐wake amounts before lights‐off, divided by those after lights‐off.

In addition to quantitative analyses, a qualitative approach consisted in assessing the possible difference of EEG power spectrum between infected and uninfected groups. EEG power spectra were computed every 5‐sec epochs for the frequency range of 0.5‐40 Hz using fast Fourier transform. For each vigilance state, the total power of densities was obtained over the frequency range of 0.5‐40 Hz for each state of vigilance by adding all epochs of a given state. All power spectral densities were standardized at the different frequency ranges, which were expressed as percentage relative to the total power observed for each vigilance state.

#### 2.4.1 Experimental procedure

We performed three sets of independent experiments to minimize the possible variation in the effectiveness of inoculation. For all sets, we simultaneously recorded infected *vs*. uninfected control mice. We used two infected *vs*. two uninfected control mice in the first, pilot batch, and two controls *vs*. six infected mice for each one of the two subsequent sets. In total, six control and 14 infected mice were subjected to the following experimental procedures.

#### 2.4.2 Spontaneous cortical EEG and sleep‐wake recordings

After recovery from surgery and habituation to the recording cables, each mouse was recorded for two days. We repeated such recording sessions two to three times a week. Our recordings could last up to six months to evidence chronic alterations in sleep/wake cycle, as the pi period may be important to reveal possible differences between control and infected mice.

#### 2.4.3 Cortical EEG and sleep‐wake cycle after pharmacological administration

Using pharmacological tools, we tentatively evaluated whether *Tg* infection causes sleep‐wake effects by affecting surrounding tissue via inflammatory process or by direct *Tg* activity or excretion. The anti‐inflammatory corticosteroid DXM (dexamethasone sodium phosphate) was administered at a dose of 2.5 mg/kg/day (5 mg DXM per liter of drinking water) for seven consecutive days.^28^ A longer period of administration has been shown to lead to a immunodepression which may reactivate *Tg*.^29^

Sulfamethoxazole‐Trimethoprim (SXT), an antimicrobial synergistic compound (Bactrim®) exhibiting activity against replicating forms of the parasite, is generally used against the effects of the acute phase of *Tg* infection. It was administered in drinking water respectively at a dose of 95 mg/kg/day and 19 mg/kg/day for 10 consecutive days.^28^

**FIGURE 1:**
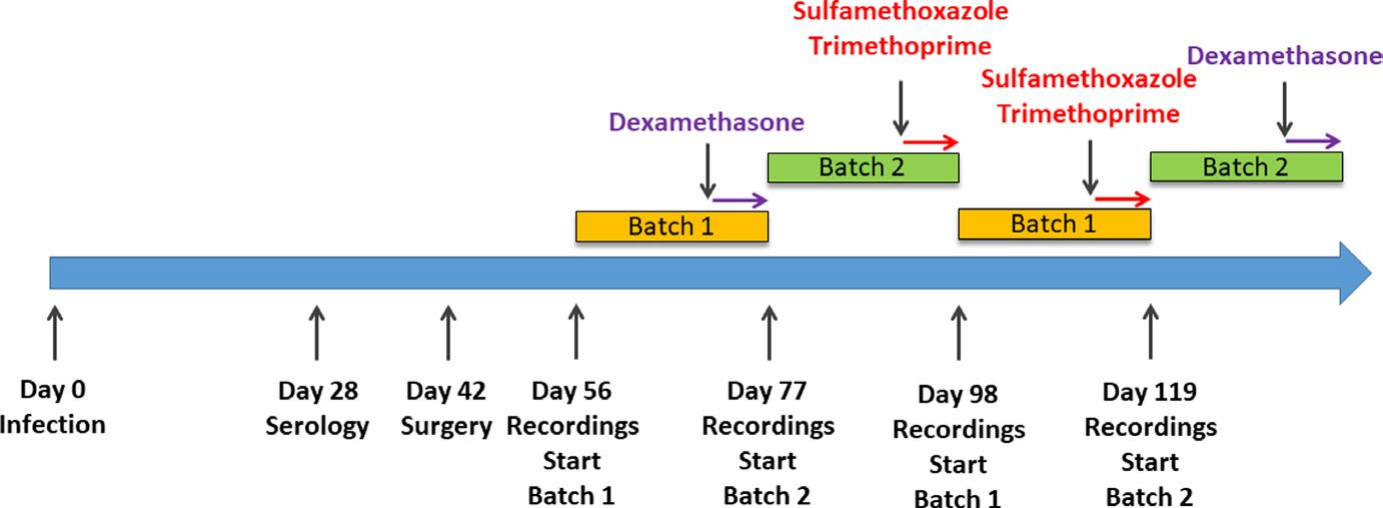
Chronology of experiments. Colored bands represent recording periods, colored arrows represent pharmacological administration of either dexamethasone or sulfamethoxazole‐trimethoprim. Time between recording periods consists of a washing period with no EEG nor EMG recordings

Treatments were separated by a wash‐out of 2 weeks (Figure 1).

### 2.5 Statistical analysis

We used GraphPad Prism 7.0 for all analyses. All experimental data were firstly examined for their value distribution using d’Agostino Pearson normality test. When the data were non‐normally distributed, nonparametric Mann‐Whitney tests were used. Two‐way ANOVA and unpaired Student's *t* test were used when the data were normally distributed. Two‐way ANOVA with repeated measures for both factors was used to evaluate the differences between control and infected mice with pi time. Drug effects were evaluated with pi time using the same procedure. These tests were followed by post hoc Holm‐Sidak test for multiple pair‐wise comparisons. All data are expressed as the mean ± SEM. All tests used were two‐tailed with a significance level of alpha set at 0.05.

## 3 RESULTS

### 3.1 General observations

**FIGURE 2:**
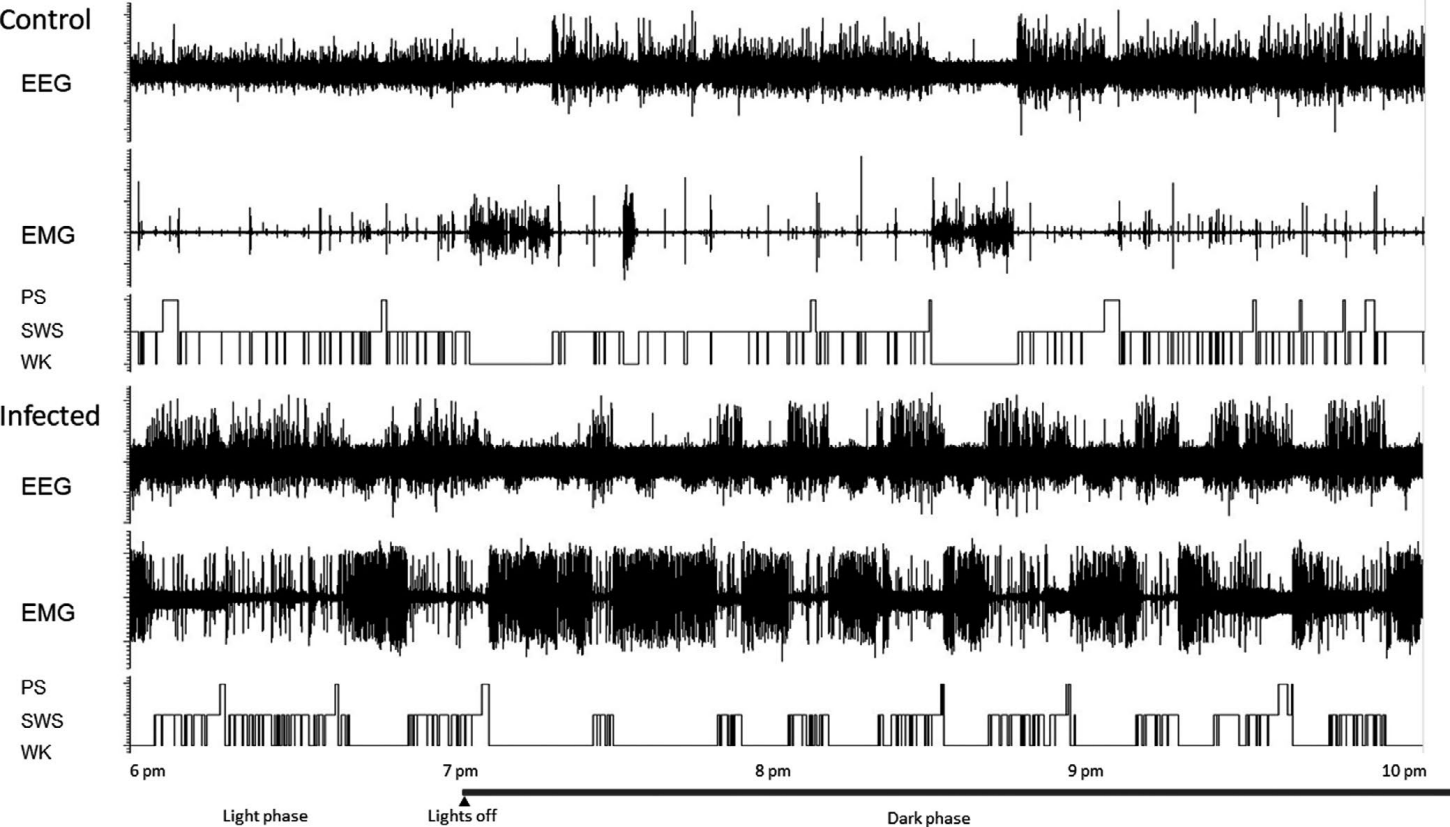
Comparison of spontaneous sleep‐wake parameters in non‐infected (up) and infected mice (low): Examples of polygraphic recordings and corresponding hypnograms showing the spontaneous sleep‐wake cycle

Infected mice appeared to develop normally as compared to uninfected mice. No obvious abnormalities were detected in terms of general morphology, food intake, reactivity when being handled, or other behaviors under baseline conditions. Yet, infected mice appeared to execute more locomotive activities than uninfected mice as shown by our electromyogram (EMG) recordings (Figure 2). As reported previously,^30^ a significant difference in body weight appeared at week 2 pi (−1.7 g weight gain vs +1.7 g in uninfected mice; n = 10 for both groups, *P *= 0.0159, nonparametric Mann‐Whitney test). No difference in lifespan was observed.

### 3.2 Increased wakefulness and decreased slow‐wave sleep in Tg‐infected mice

**FIGURE 3:**
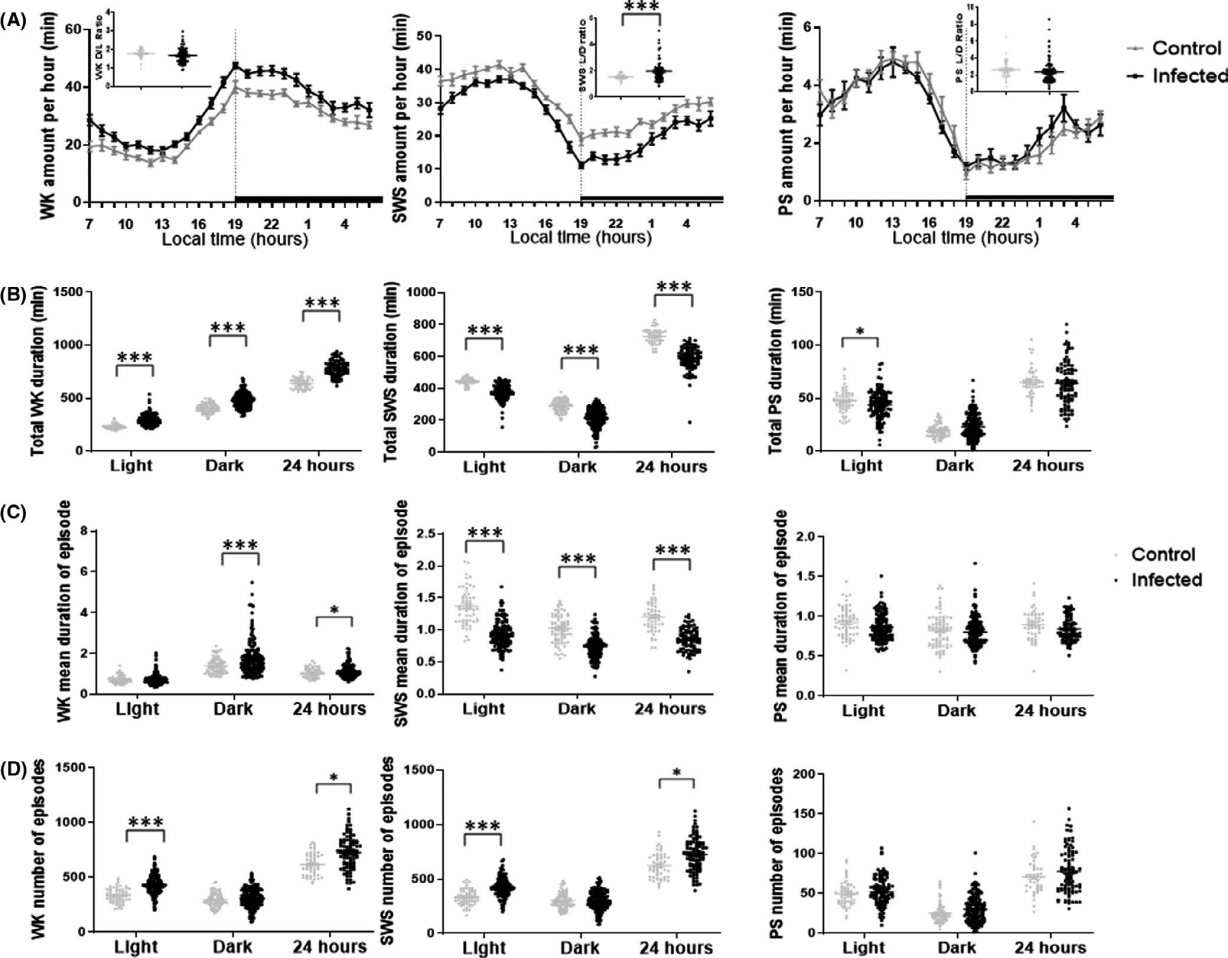
A, Sleep‐wake states amount per hour: Mean hourly values (± SEM in min) of the sleep‐wake states. The dark areas correspond to the dark period. The non‐background inserted dot plots correspond to the light/dark or dark/light (L/D or D/L) ratio of sleep‐wake amount before and after lights‐off. B, Total sleep‐wake states duration: Means ± SEM (in min) of the sleep‐wake stages for the 12 h light and dark and 24 h periods. C, Mean duration of episodes of each sleep‐wake state during the light, dark and 24 hours period (mean values ± SEM (in min)). D, Mean number of episode of each sleep‐wake state during the light, dark, and 24 hours period (mean values ± SEM). Other abbreviations: PS, Paradoxical Sleep; SWS, Slow‐Wave Sleep; WK, Waking. *P<0.05,***P<0.0001 two‐way ANOVA and unpaired Student's *t* test (A, inserted dot plots, two tails)

In baseline conditions, *Tg* mice exhibited a circadian sleep‐wake rhythm quite characteristic of CBA/Jrj mice, with increased activity (Figure 2) and a larger amount of waking (WK) during the dark phase (Figure 3). Compared to uninfected mice, the total amount of WK) in *Tg* mice was significantly increased over all light‐dark phases. This effect was due to a significant increase in both mean episode duration and number of episodes during 24 hours, with a marked increased episode duration during the dark phase and a more pronounced increase in the number of episodes during the light phase (Table 1).

**TABLE 1:** Summarized results for difference in vigilance states (total amount, number of episodes, and duration of episodes) between infected and control mice

	24 hours			Light period			Dark period		
	Infected	Control	*P*	Infected	Control	*P*	Infected	Control	*P*
WK									
Mean total amount	785.7±8.747	634.8±7.629	<0.0001	298.2±4.732	231.7±3.526	<0.0001	478.5±5.458	401.8±5.514	<0.0001
Mean number of episodes	727±15.33	625.9±16.35	0.0106	429.1±7.907	332.3±8.752	<0.0001	306.9±6.744	301.6±7.801	0.4312
Mean duration of episodes	1.145±0.03537	1.061±0.03358	0.0260	0.7435±0.02306	0.7262±0.02307	0.2377	1.794±0.07682	1.416±0.04897	<0.0001
SWS									
Mean total amount	586.6±7.907	730.9±7.2	<0.0001	378.0±4.384	439.7±3.43	<0.0001	215.9±4.764	296.5±4.938	<0.0001
Mean number of episodes	709.1±14.97	629.1±17.39	0.0268	422.4±7.706	332.8.9±8.944	<0.0001	300.5±6.756	301.2±7.949	0.2258
Mean duration of episodes	0.8535±0.01778	1.207±0.03206	<0.0001	0.9272±0.01804	1.385±0.04438	<0.0001	0.7411±0.01504	1.027±0.02565	<0.0001
PS									
Mean total amount	64.1±2.150	68.1±3.157	0.4055	43.6±1.188	48.4±1.739	0.0059	23.0±0.974	20.8±1.172	0.8047
Mean number of episodes	78.14±2.940	76.69±4.236	0.7743	52.0±1.578	57.2±4.781	0.0711	30.09±1.309	29.63±3.499	0.7574
Mean duration of episodes	0.8427±0.0158	0.8990±0.02594	0.5077	0.8624±0.01439	0.9227±0.02329	0.5709	0.8028±0.01377	0.8228±0.02373	0.9429

Note

Figures represent mean values ± SEM (minutes) for Tg‐infected mice and ‐uninfected mice (control) during the different periods (24 hours, light, and dark). *P* value is calculated using two‐way ANOVA with repeated measures to evaluate the differences between control and infected mice. All tests used were two‐tailed with a significance level of alpha set at 0.05.

Concomitantly, the total amount of slow‐wave sleep (SWS) was decreased over all phases (*P *< 0.0001). The decreased SWS was characterized by a decrease in episode duration and an increase in episode number (Table 1) indicating sleep fragmentation in *Tg* mice. Such sleep fragmentation occurred more prominently during the light phase (the primary sleep period) than during the dark phase (when mice are more aroused) (Table 1). No fragmentation was noted with regards to WK. As revealed by hourly analysis of the sleep‐wake amounts, the increase in WK and decrease in SWS were greater during the dark phase than during the light phase and notably during the first 4‐5 hours immediately after lights‐off. The larger change of SWS during the dark phase leads to an increased light/dark ratio for SWS from 1.53 to 1.98 (*P *= 0.0002) (Figure 3), indicating disturbed circadian sleep rhythms in *Tg* mice. Nevertheless, WK light/dark ratio had no significant change. When analyzing latencies to sleep after lights‐off, we found a significant delayed occurrence for SWS in *Tg* mice: 1169 s vs. 465 s (nonparametric Mann‐Whitney test, *P *= 0.0001), in consistence with the enhanced WK observed with *Tg* mice.

The decrease in SWS was accompanied by a reduced total amount of paradoxical sleep (PS) during the light phase only (*P* = 0.0059) (Table 1, Figure 3). No significant changes of PS, either in terms of total amount or episode number and duration, were noted during the dark phase and over 24 h. The latency to PS after lights‐off was not significantly modified (4638 s *vs*. 4608 s; *P* = 0.8412).

### 3.3 Time course of sleep‐wake changes induced by Tg infection

**FIGURE 4:**
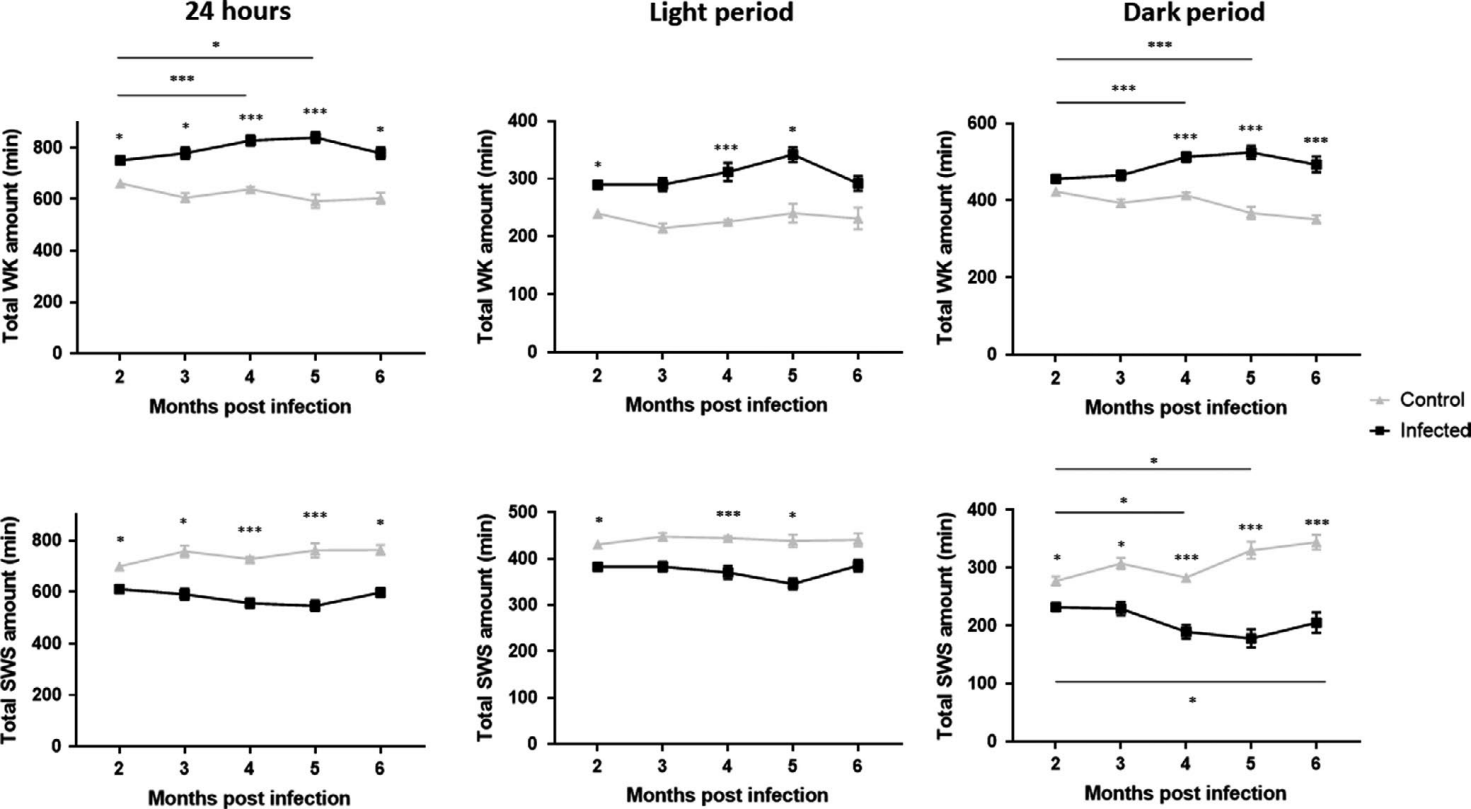
Influence of the time since infection on WK and SWS durations between infected (black) and control (uninfected) mice (gray), means ± SEM (in min) of the sleep‐wake stages for the 12 h dark and 24 h periods, showing an increase of WK with a decrease of SWS over time since infection. Time exacerbates the effects of Tg infection for WK total durations (dark: p_interaction Tg/time_<0.0001, 24 hours: p_interaction Tg/time_ = 0.0013), as well as for SWS total duration during the dark period (p_interaction Tg/time_<0.0001) and over 24 hours (p_interaction Tg/time_ = 0.011). Other abbreviations: Tg, *Toxoplasma gondii*; PS, Paradoxical Sleep; SWS, Slow‐Wave Sleep; WK, Waking. *P<0.05, ***P<0.0001 two‐way ANOVA followed by Holm‐Sidak multiple comparison post hoc tests

The increase in WK and decrease in SWS seen with *Tg* mice occurred in the second month. The effects were amplified during the third and fourth month, reaching their maximum at the fifth month (Figure 4), and remained important and statistically significant at the sixth month. The impact of time from infection was further confirmed using two‐way ANOVA for both WK and SWS (Figure 4), on their total mean duration (*P* = 0.008 and *P* = 0.0209 for 24 h and light phase WK amount; *P* = 0.0395 and *P* = 0.0002 for 24 h and light phase SWS amount) and on their mean number of episodes during 24 h, light and dark periods for WK, and during the dark period for SWS. Finally, we examined the effect of *Tg* infection on the time course of these sleep‐wake changes with the interaction term of ANOVA and found that they were modified according to the infective status. Indeed, *Tg* mice showed an increased WK and a decreased SWS over time whereas uninfected mice exhibited a decreased WK and an increased SWS (Figure 4). Thus, time exacerbates the effect of *Tg* infection on the sleep‐wake states.

In contrast, PS amount during all light‐dark phases had no clear change across time (data not shown).

### 3.4 Qualitative alterations in power spectral density of cortical EEG during sleep‐wake states

**FIGURE 5:**
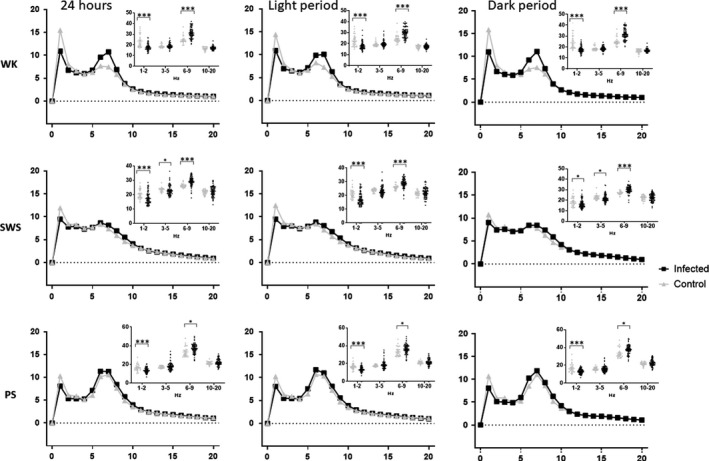
Mean spectral distribution of cortical EEG power density (X axis in Hz) in baseline sleep‐wake states in infected and control (uninfected) mice for each state (WK line 1, SWS line 2, PS line 3) and each period (24 hours column 1, light column 2, and dark column 3). The data were obtained by pooling consecutive 5 s epochs during 24 hours using the fast Fourier transform routine within the frequency range of 0.5‐20 Hz. For each curve, cortical EEG power band density (dot plot) is represented for infected (black) and control (gray) mice. Note that infected mice show an increase in theta band (6‐10 Hz) during all states and a decrease in slow activity in 1‐2 Hz range during all states. Other abbreviations: PS, Paradoxical Sleep; SWS, Slow‐Wave Sleep; WK, Waking. *P<0.05, ***P<0.0001 nonparametric Mann‐Whitney test (two tails)

The distribution of cortical EEG power density and their spectral morphology during all light‐dark phases were quite similar between *Tg* and WT mice. Yet, we noticed a markedly increased power spectral density in the θ rhythm (6‐9 Hz) for all sleep‐wake states, notably WK, as well as a decrease in low δ rhythm (1‐2 Hz) (Figure 5). Of note, the differences seen during PS were less important than those seen during SWS and WK.

### 3.5 Effects of pharmacological administrations on the sleep‐wake changes caused by Tg infection

Since the major effect of *Tg* infection was enhanced WK, we attempted to identify its possible origin. Notably, such an effect could be due to a direct aggression on the brain tissue by *Tg* cyst or tachyzoite or to inflammatory reactions around the infected loci causing such long‐lasting WK enhancement. Data were analyzed globally over the whole course, that is, from the first to the last days of both treatments to take into account possible mechanisms of compensation. However, we observed the same effects on all periods (light, dark, and 24 h periods).

Firstly, we administered the anti‐*Tg* compound SXT and found that its chronic oral application via water supply had no significant effects on the ongoing sleep‐wake states in either uninfected or infected mice. Thus, the enhanced WK and decreased SWS seen in *Tg* mice remained unchanged. No major effects were seen on the cortical EEG except in uninfected mice: a slight increase in the low δ rhythm (1‐2 Hz) during light phase SWS (*P*<0.0001). The cortical EEG changes seen with *Tg* mice, that is, the increase in power spectral density in the θ rhythm and the decrease in low δ rhythm (1‐2 Hz) for all the vigilance states remained virtually identical during and after the SXT treatment. Thus, SXT had little effect on the EEG and sleep‐wake changes caused by *Tg* infection under the current experimental conditions.

**FIGURE 6:**
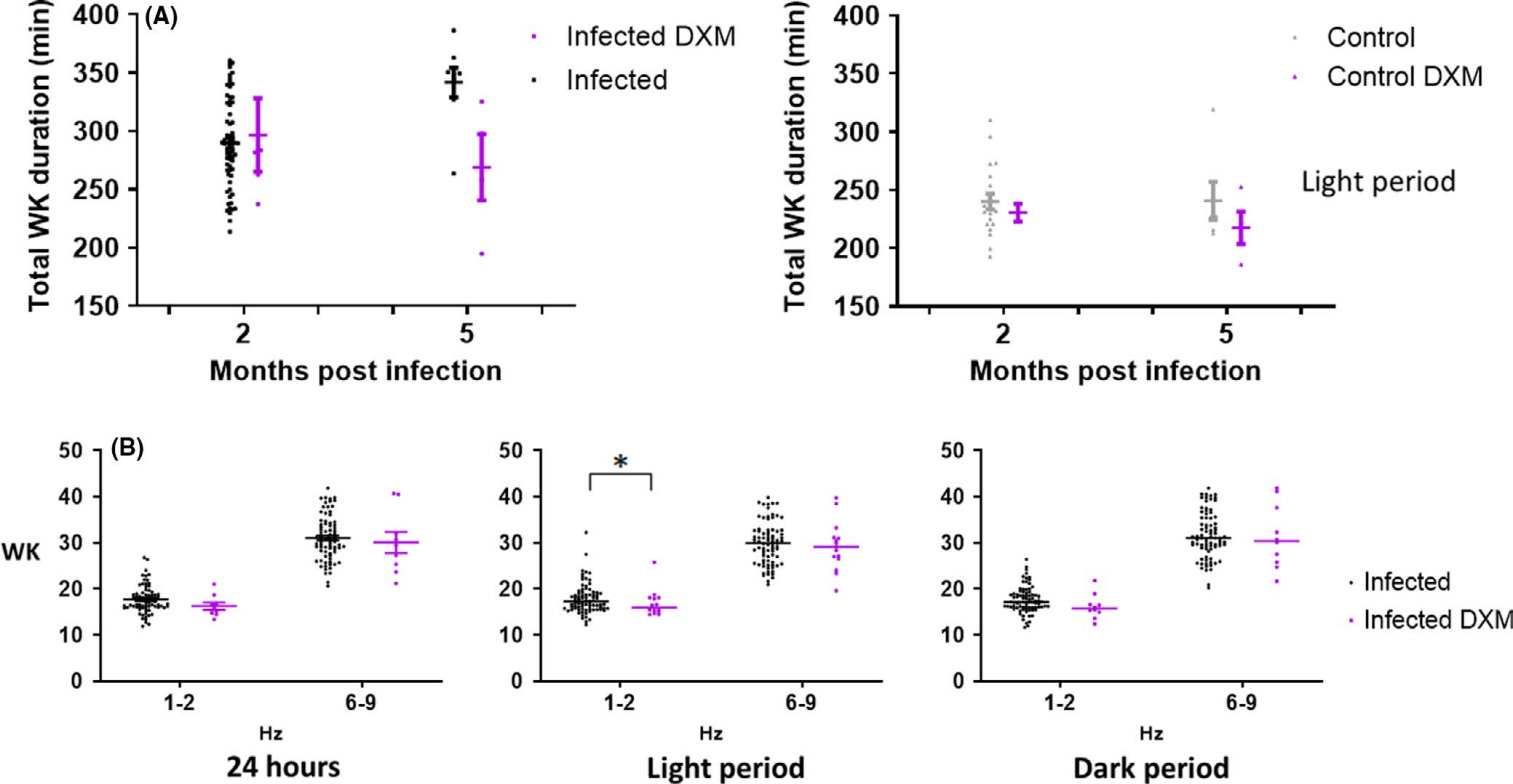
A, Influence of the time since infection on WK durations between infected (black) and infected mice treated with DXM (purple), means ± SEM (in min) of the sleep‐wake stage for the 12 h light period, showing that increased WK due to infection is alleviated by DXM. This effect is impacted by time since infection. B, Relevant cortical EEG power band density (dot plot) is represented for infected (black) and infected+DXM (purple) mice during WK, vigilance state in which we observed major quantitative changes. Other abbreviations: pi, post‐infection; WK, Waking. *P < 0.05,***P < 0.0001 A/two‐way ANOVA followed by Holm‐Sidak multiple comparison post hoc tests and B/ nonparametric Mann‐Whitney test (two tails)

Conversely, administration of the anti‐inflammatory corticosteroid Dexamethasone (DXM) affected the sleep‐wake changes produced by *Tg* infection. Indeed, chronic oral application via drinking water notably during the fifth month of the infection, where the maximal enhanced WK was observed (Figure 6), resulted in a significant decrease in WK amount during the light phase in the treated *Tg* mice as compared with the untreated ones (298.2 ± 4.73 *vs*. 284.4 ± 20.82; *P* = 0.04, p_interaction DXM/time_ = 0.01). Despite the decreased WK effect, the increase in power spectral density in the θ rhythm and the decrease in low δ rhythm (1‐2 Hz) for all the vigilance states, identified in the *Tg* mice, remained similar or were slightly enhanced during and after the DXM treatment. Nevertheless, a moderate decrease in the power of low δ rhythm during the light period (1‐2 Hz) for WK (*P*<0.0001) was observed (Figure 6).

Since DXM likely counteracted some effects of *Tg* infection, we performed a few additional control tests (i) administration of DXM at the 2nd month of the infection where the enhanced WK just became highly significant: There were no significant effects on the enhanced WK indicating time course‐specific effect of DXM (*P* = 0.022); (ii) administration of DXM in control uninfected mice: a slight and statistically non‐significant decrease in WK was noticed (Figure 6), indicating that the counteracting effect of DXM on WK was specific to *Tg* infection.

In summary, DXM alleviated sleep‐wake alterations due to *Tg* infection by counteracting the enhanced WK quantitatively, while having no qualitative effect on cortical EEG, indicating involvement of inflammatory processes, somehow, in the increased amount of WK associated with *Tg* infection.

## 4 DISCUSSION

### 4.1 A chronic murine model of Tg infection and experimental considerations

Sleep‐wake impairment is often associated at an early stage with mood, behavioral, and neurological disorders.^15^, ^16^ Putative involvement of *Tg* infection in this association has been suggested by indirect evidence ^12^, ^31^, ^32^, ^33^ and prompted us to study the effects of latent, chronic *Tg* infection on sleep‐wake cycle, an overall indicator of the impact of *Tg* on cerebral functions.

For this purpose, we designed an ecological model allowing us to control all aspects of *Tg* infection over time, and to perform in depth analysis of wake‐sleep cycle including EEG power spectral analysis in experimental conditions. We used the Prugniaud strain whose lower virulence and kystogenic profile are advantageous for analyzing the CNS consequences of chronic toxoplasmosis. We are not aware of similar experiments. The few previous studies addressing the question in patients were limited by the use of indirect outcomes such as sleep apnea or daytime sleepiness,^34^ or sleep self‐report,^35^ and by the need to rely on a single anti‐*Tg* IgG test to recognize infected subjects, precluding any estimation regarding time elapsed since infection.

Schematically, the parasite affects the brain by means of two major components: direct parasitic effect or inflammation in response to the parasite aggression. We tried to address this issue by studying pharmacologically the impact of an anti‐parasitic or anti‐inflammatory compound on the induced sleep‐wake alterations. Considering the fact that SXT has little effect on cysts as opposed to tachyzoites, the active disseminating form of the parasite, we nonetheless chose to test SXT since Watts et al demonstrated that cysts were in fact active and replicating.^36^ On the other hand, we treated mice with Dexamethasone (DXM) seven days in order to access the involvement of inflammation while preventing reactivation of cysts: This length of treatment was reported not to promote sleep‐wake alterations in rats.^37^

Finally, the presence of blood‐born anti‐*Tg* antibodies in our *Tg* mice attests the efficiency of Tg infection and supports it as origin of the sleep‐wake alterations.

### 4.2 A long‐lasting, marked and rarely observed enhancement of wakefulness

The main original finding of our study is that chronic *Tg* infection significantly alters the sleep‐wake cycle in the mouse. These alterations consisted in a markedly increased amount of wakefulness, particularly during the dark period. SWS was concomitantly decreased at all periods. We found that the anti‐*Tg* drug SXT had no clear impact on the sleep‐wake effects after *Tg* inoculation, presumably because the direct impact of the parasite at this late chronic stage (ie, > month 3 pi) was already stable and irreversible. In contrast, the corticosteroid anti‐inflammatory DXM reversed the increased WK quantitatively but not qualitatively. These pharmacological data seem to be quite conclusive for the involvement of an inflammatory process in the *Tg*‐induced WK enhancement rather than that of the parasite itself.

Our results are in line with previous findings regarding *Tg* infection. First, sleep is an orchestrated neurochemical process on different brain networks^38^ and involves many neurotransmitters and modulators, notably monoamines, whose balance was found to be modified after *Tg* infection. Indeed, *Tg* infection increases the level of dopamine but not that of serotonin and norepinephrine,^22^ indirectly via the release of inflammatory cytokines, but mostly directly via the presence in its genome of genes coding for phenylalanine hydroxylase and tyrosine hydroxylase.^39^ Second, as a consequence of IFN‐alpha‐driven host's response, *Tg* infection induces the degradation of tryptophan required for the parasite growth into kynurenine neuroactive metabolites, via indoleamine 2,3‐dioxygenase. Such a catabolic shunt of tryptophan, precursor for serotonin and melatonin, may alter their turnover,^20^, ^21^ thus altering sleep and circadian rhythm.^40^ Finally, several brain sleep‐regulating structures ^41^, ^42^, ^43^ have been reported to be more infected, for example, the amygdala, cortex, brainstem, olfactory bulb, and hippocampus.^18^, ^19^

Although a moderate SWS fragmentation was noted during the light phase, primary sleep period in mice, this effect was not associated with WK fragmentation and did not extend to the dark phase, when mice are more awake and active. Thus, this slight sleep fragmentation was not associated with any sign of somnolence or sleepiness like in many sleep disorders and could instead be regarded as weakened SWS maintenance, consistent with the enhanced WK. Moreover, we found in infected mice that EEG θ power, a sign of cortical activation, increased during the enhanced WK for all light‐dark phases and that latency to SWS was delayed after lights‐off. All these findings underline a high level of generalized alertness in infected mice. Intriguingly, this enhanced WK and alertness increased with time and lasted over several months, which is very rarely observed with any experimental model or protocol designed to enhance WK.^44^, ^45^, ^46^ Most surprisingly, sleep loss elicited by such long‐lasting WK enhancement appeared to be well tolerated by *Tg* mice. Indeed, sleepiness, sleep rebound, or compensating increased cortical slow activities during SWS, which could be expected, was not seen at all during any light‐dark phase. Mice were generally active as seen with our EMG recordings. Their morphology, food intake, and lifespan had no apparent changes as compared to uninfected animals. Moreover, a decreased locomotion and increased body weight, often associated with chronic sleep restriction,^47^, ^48^ were not identified with *Tg* mice. Instead, their body weight decreased slightly. These results suggest that *Tg* mice maintain their homeostatic integrity and could serve as a model of chronic sleep restriction. No change of the light/dark ratio for WK but a mild increase for SWS was noticed indicating a limited effect on circadian balance despite such a marked increase in WK.

**FIGURE 7:**
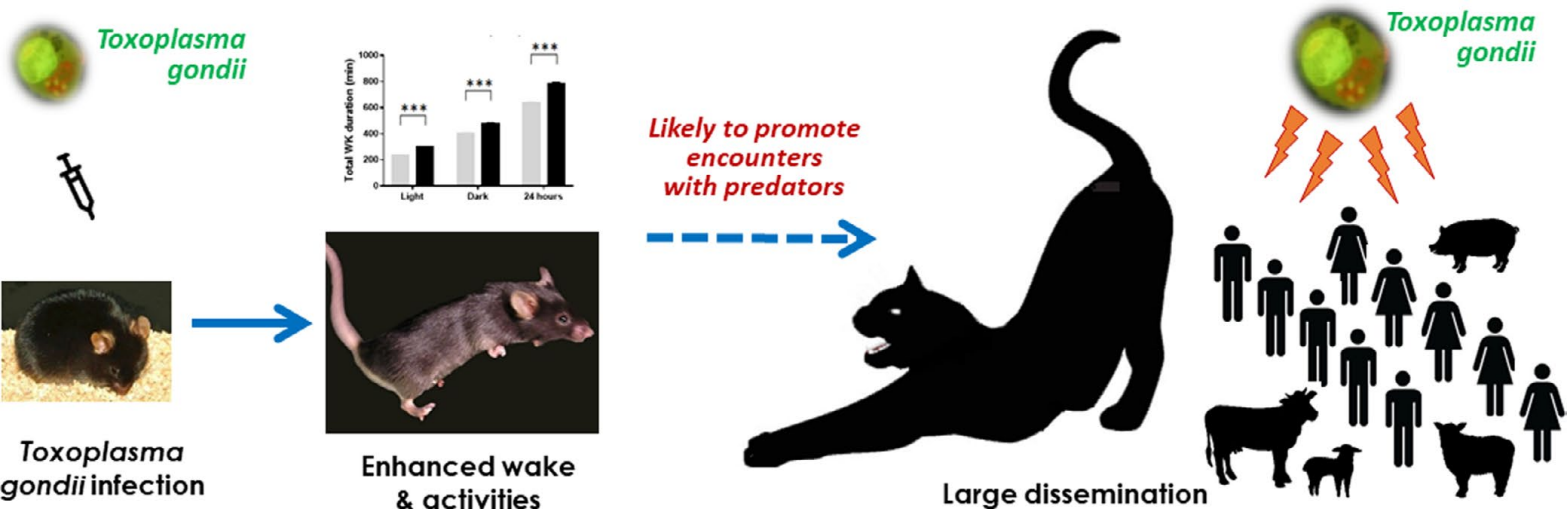
Graphic abstract illustrating the main findings of the present study and our interpretations. When mice are infected chronically by *Toxoplasma gondii*, an intracellular parasite, they exhibit a marked increase in wakefulness and activities, thus promoting encounters with cats, its natural predators. It is likely that by this strategy to modify its hosts’ sleep‐wake and behavioral states, *Toxoplasma gondii* would enhance its own dissemination, thus explaining why it infects a wide range of animals and about 1/3 of the world population

While the neural mechanisms underlying this remarkable WK enhancement and “well‐being” of *Tg* mice remain to be investigated, the *Tg* infection model established here would explain, at least partly, the large prevalence of *Tg* infection in animals and humans (see graphical abstract in Figure 7).

### 4.3 A putative strategy of Tg to promote its dissemination

This persistently increased WK and reduced sleep in mice with chronic *Tg* infection have not been previously reported but fit well with its suspected strategy by which it would promote its own dissemination by enhancing WK in order to facilitate encounters with their predators.^8^, ^49^ Increased WK was mostly noticed during dark phases when increased activity is more likely to favor predation of nocturnal animals like mice. Reduced sleep duration at all phases would also contribute to keeping animals more opportune (Figure 7). Sleep and WK highly impact vigilance and attention processes. Although a lower attention and longer reaction time have been reported in *Tg*‐infected animals^50^, ^51^ and man,^52^, ^53^, ^54^ their links with WK alteration were not reported. The impact of this WK enhancement on vigilance and attention remains therefore to be addressed with specific behavioral tests. Since the enhanced WK was stable over the whole periods, it would last for the host's entire lifespan, as a permanent outcome of infection. Intriguingly, they were not immediately at their strongest. Such progressive increase with the time elapsed pi was also reported in humans: the intensity of personality trait shift correlates with estimated duration of infection.^55^ Manifestations of delayed sleep onset and restless legs syndromes that were observed in our patients with postnatal or congenital *Tg* infection tend to develop at least six to 10 years pi (Lyon cohort).

Considering sleep‐wake cycle as a major brain function targeted by *Tg* to manipulate the behavior of infected hosts might also explain why our results are contrasted with other infection models that tend to report an increased sleep duration, suspected to be the consequence of increased interactions between inflammatory cytokines and brain sleep‐regulating structures. This was the case for acute systemic infections,^56^ including influenza,^57^ but also in chronic infection. Herpes virus is reported to be associated with increased sleep duration and fragmentation.^58^, ^59^, ^60^

### 4.4 Medical significances and perspectives

We believe that the combination of parasite/host (Prugniaud/male CBA) satisfies most of the criteria of latent infection as seen in humans. Nonetheless, further investigations are required to assess whether our animal model can extend to females and other strains, as they may differ on their susceptibility to the acute or chronic phase of infection depending on their HLA haplotype.^61^ It is also important to extend our study to congenitally infected mice, as the effects of congenital infection are generally more pronounced, exhibiting decreased cognitive capacities and increased anxiety compared to mice infected after birth.^51^

In particular, the role of monoamines would deserve a central attention given their marked alteration by *Tg* infection (34‐36), and their involvement in mood control, motor activity, and attention functions also targeted by *Tg*. Our novel model together with the knockout *Tg* construct designed by Wang et al (deleted of one copy of Tyrosine Hydroxylase^62^) would constitute promising tools not only with this purpose, but also in pharmacological assessment testing the potential anti‐*Tg* therapies to normalize behavioral activities and to alleviate psychiatric symptoms in infected patients.

Finally, given the large prevalence of *Tg* infection, any new finding regarding *Tg*‐associated sleep‐wake regulation would represent a considerable medical and socioeconomic impact. Our results, if clinically confirmed, would also promote serological or molecular detection of *Tg* in patients suffering from sleep disorders and prevention campaign against toxoplasmosis, the importance of which is sometimes debated. Such possible underestimation of risk associated with *Tg* infection may also concern the current worldwide debate on the cost‐effectiveness of prenatal screening programs.

Altogether, we have established a novel, currently unique and insightful murine model of *Tg* infection suitable for basic and translational investigations. The use of this model has allowed us to demonstrate the *Tg*‐induced sleep‐wake alterations, notably a remarkable long‐lasting enhancement of WK, with which *Tg* would likely promote its own propagation. Our study also demonstrates sleep‐wake assessment as a potent and pertinent approach for long‐term follow‐up of infectious events, that is not always expected or sought.

## CONFLICT OF INTEREST

The authors declare no competing financial interests.

## AUTHORS’ CONTRIBUTION

DD involved in conceptualization, data curation, formal Analysis, investigation, methodology, project administration, validation, visualization, writing—original draft, and writing—review and editing. JSL involved in project administration, resources, supervision, validation, and writing—review and editing. FP involved in writing—review & editing. HA involved in conceptualization, data curation, formal Analysis, investigation, methodology, project administration, resources, supervision, validation, visualization, writing—original draft, and writing—review and editing. MW involved in conceptualization, project administration, resources, methodology, supervision, validation, and writing—review and editing.

## Data Availability

Data available on request from the authors.
